# Regulatory T cells and M2 macrophages present diverse prognostic value in gastric cancer patients with different clinicopathologic characteristics and chemotherapy strategies

**DOI:** 10.1186/s12967-019-1929-9

**Published:** 2019-06-07

**Authors:** Xu Liu, Danhua Xu, Chen Huang, Yixian Guo, Shuchang Wang, Chunchao Zhu, Jia Xu, Zizhen Zhang, Yanying Shen, Wenyi Zhao, Gang Zhao

**Affiliations:** 1 Department of Gastrointestinal Surgery, RenJi Hospital, School of Medicine, Shanghai Jiao Tong University, 160 Pujian Road, Pudong New Area, Shanghai, 200025 China; 2 Department of Pathology, Renji Hospital, School of Medicine, Shanghai Jiao Tong University, 160 Pujian Road, Shanghai, 200025 China

**Keywords:** Gastric carcinoma, Prognosis, FOXP3^+^ regulatory T cell, M2 macrophage, Subgroups

## Abstract

**Background:**

Gastric cancer (GC) remains a refractory cancer worldwide. Currently, exploring the differences of the immune status in GC patients with different subgroups might provide promising immunotherapeutic approaches for the treatment of GC.

**Methods:**

In this study, a total of 598 surgically resected FFPE primary gastric cancer samples were assessed for FOXP3, CD163, CD3, CD8, and PD-L1 markers. The correlations between the immune markers expression and clinicopathological features and prognosis were investigated retrospectively.

**Results:**

In general, PD-L1, CD3, and CD8 could be regarded as favorable prognostic factors. Our data demonstrated that high infiltration of FOXP3^+^ Treg indicates better prognosis in stage I–II patients, while the converse outcome was noted in stage III–IV patients. Our data also confirmed different prognostic value in different pathological classifications, chemotherapy strategies, and locations, with or without lymph node metastasis. Also, M2 macrophages indicated poor prognosis in general. However, high M2 macrophage infiltration suggests a favorable prognosis in signet ring cell carcinoma and mucinous adenocarcinoma. Moreover, the prognostic value of the two indices when they are combined is reported.

**Conclusions:**

These results suggested that different immune statuses are exhibited in different subgroups of GC, which may direct further understanding of the immune status of GC as well as provide a further theoretical basis and potential targets for GC immunotherapy.

**Electronic supplementary material:**

The online version of this article (10.1186/s12967-019-1929-9) contains supplementary material, which is available to authorized users.

## Background

Gastric cancer (GC) remains a refractory cancer worldwide, and it still ranks the fifth most common cancer and the third leading cause of cancer death [1]. According to global cancer survival data of GC for 2000–2014, the 5-year survival rate of Chinese GC patients was only 35.9% [2]. The typical malignant behavior of GC involves lymph node (LN) metastasis [3]. At the time of diagnosis, 35% of GC patients exhibit evidence of distant metastases, and distant metastasis is commonly considered to be a invariably fatal situation in GC [4]. Although the infiltrating status of some immune cells has been reported in different locations of GC [5], there are limited reports about specific immune cells with different indications for prognosis in different locations of GC. The World Health Organization (WHO) histological classification is widely accepted and used for the diagnosis in GC, and the Japanese classification divides the common types of gastric adenocarcinoma into additional subtypes, which is widely used [6, 7]. Some studies have described poor prognosis for the signet ring cell carcinoma and mucinous adenocarcinoma compared with other subtypes of GC [8, 9].

With GC progression, the infiltrating status of specific immune cells and their differences in predicting prognosis have not been discussed at length in different pathological classifications of GC. Given that GC is a complex disease with heterogeneity [10], subgroup analysis is necessary to further improve the accuracy of immune indicators for the prognosis of GC patients.

Chemotherapy with platinum and fluoro-pyrimidine is currently the recommended first-line therapy for patients with advanced or metastatic GC who have good performance status [11]. In recent years, immunotherapy with immune checkpoint inhibitors has revolutionized the treatment of advanced cancer and demonstrated quite promising results [12–14]. Blockade of the PD-L1/PD-1 pathway remains ineffective in GC [15, 16]. Hence, to enhance the efficacy of immunotherapy in GC, identifying some more precise predictive biomarkers in order to select better GC patients who might benefit most from immunotherapy is urgently required. Recently, a positive correlation between the presence of tumor-infiltrating lymphocytes (TILs) and survival of patients with malignancies was reported [5]. High infiltration of CD3^+^ and CD8^+^ T lymphocytes is associated with better outcome in GC, demonstrating the critical role of T cell-mediated host immunity in repressing tumor progression [17, 18]. Tumor infiltrative forkhead box P3-positive (FOXP3^+^) regulatory T cells (Tregs) might play a crucial role in the immune microenvironment of GC [19–21]. However, the prognostic value of Treg infiltration remains controversial in GC patients [22, 23]. Also, tumor-associated macrophages (TAMs) are known to directly or indirectly support the tumor growth and metastasis in GC and are positively associated with the depth of invasion and clinical stage. TAMs display high plasticity and perform diverse, supportive functions which were specialized for different tissue compartments [24, 25]. M2 macrophages are associated with promoting tumor growth, remodeling tissue, promoting angiogenesis, and suppressing adaptive immunity. CD163, a member of the scavenger receptor cysteine-rich family, is a M2 macrophage marker [26, 27].

In this study, we used a tissue microarray (TMA) including 598 GC primary lesion specimens to investigate the expression of PD-L1 and quantify tumor-infiltrating CD3^+^, CD8^+^ T lymphocytes, FOXP3^+^ Tregs, and CD163^+^M2 macrophages density to determine their relationships with clinicopathological features and prognosis in GC patients based on different locations, clinicopathological stages, pathological classifications, chemotherapy strategies, and lymph node metastasis. Compared with previous relevant studies, our research have conducted an in-depth analysis of different subgroups of GC, which have rarely been reported before.

## Methods

### Patients

This study retrospectively evaluated 598 gastric cancer samples from patients who underwent primary tumor resection between June 2005 to December 2015 at the Department of Gastrointestinal Surgery, RenJi Hospital, School of Medicine, Shanghai Jiao Tong University. All the samples were definitely diagnosed as gastric cancer by Department of Pathology. We excluded the following types of patients: (1) patients without complete clinical information, postoperative pathological diagnosis, follow-up data, etc.; (2) patients receiving previous radiotherapy or other neoadjuvant treatment; (3) patients with non-neoplastic resection, such as laparotomy or palliative gastrointestinal bypass surgery, and non-adenocarcinoma patients, such as those with gastrointestinal stromal tumors, etc.; (4) patients who suffered from other primary malignant tumors; (5) patients with perioperative death from surgical complications; (6) primary tumor involving ≥ 2 regional sites at the site of occurrence; (7) unclear pathological types; (8) tissue samples were unavailable for TMA. Overall survival time was defined as the interval between gastrectomy and either patient death or the last follow-up. The final follow-up date was June 25, 2018, for all cases examined [28–30].

All patients received standard treatments, such as D2 radical resection and adjuvant chemotherapy according to the NCCN guidelines. The first-line chemotherapy is platinum and fluorouracil. When the patient relapses, the second-line chemotherapy regimen paclitaxel is added. Among them, fluorouracil includes 5-fluorouracil or S-1 (or xeloda); cisplatin includes cisplatin or oxaliplatin; paclitaxel includes paclitaxel and docetaxel.

The patients’ tumor staging was in accordance with the American Joint Committee on Cancer (AJCC 8th edition) staging system. For each case, the diagnosis was confirmed by a senior pathologist through a review of H&E-stained slides.

### Specimens and quantitative immunohistochemical analysis

The formalin-fixed, paraffin-embedded (FFPE) tissue samples were sliced in consecutive, 5-μm-thick sections. Slides were dewaxed in xylene and rehydrated in graded ethyl alcohol before immunohistochemical staining. Immunohistochemical staining was performed based on the manual of Dako REAL EnVision Detection System (K5007, Dako). The following primary antibodies were used:

Anti-PD-L1 (1:100, 22C3, Dako); Anti-CD163 (1:100, ab87099, Abcam); Anti-CD3 (1:100, ab16669, Abcam); Anti-CD8 (1:100, ab4055, Abcam); and Anti-FOXP3 (1:100, ab20034, Abcam).

In brief, after tissue sections were deparaffinized and rehydrated with graded ethanol, tissues were incubated with 0.3% hydrogen peroxide for 30 min and then blocked with 10% BSA (Sangon, Shanghai, China). Slides were first incubated using the specific antibodies at 4 °C overnight and then labeled with the HRP secondary antibody (Thermo Scientific, US) at room temperature for 1 h. Positive staining was visualized using diaminobenzidin (DAB) substrate liquid (Gene Tech, Shanghai), and counterstained by hematoxylin.

All the staining samples were visualized using the ZEISS Axio Vert.A1 microscope system. For each sample, 5 visual fields showing the highest infiltrating densities at 40× magnification were chosen first, and cell numbers were counted at 200× magnification. After counting the cells, cell destiny was calculated as mm^2^ for further statistics. Immune cells in vessels, submucosal lymphatic areas, and necrosis/necrosis adjacent areas were not counted in this research. The positive cell numbers were independently counted, and clinicopathologic data were blinded. In a two-category immunoscore analysis, patients were dichotomized into the high- and low-density group according to the median number of stained cells. As a result, the cutoff for FOXP3 was 11/mm^2^, CD163 was 20/mm^2^, CD3 was 47/mm^2^, CD8 was 17/mm^2^. PD-L1 positivity was defined as staining in 1% or more tumor cells [31–33]. PD-L1 expression on tumor cells instead of stroma was immunohistochemically analyzed by an experienced pathologist. Intensity and percentage of stained cells were evaluated separately for tumor and immune cells by 2 individuals who were blinded to the clinicopathological data.

### Ethics statement

All experiments were approved by the Ethical Committee of the Shanghai Jiao Tong University School of Medicine, Renji Hospital, and written informed consent was obtained from patients. All procedures followed were by the ethical standards of the responsible committee on human experimentation and the Helsinki Declaration of 1964 and later versions. Informed consent or a substitute for it was obtained from all patients included in the study.

### Statistical analyses

SPSS 23.0 and GraphPad Prism 6.0 were used for statistics in this research. Overall survival analysis was performed using the Kaplan–Meier method and the long-rank test. Univariate and multivariate prognostic analyses were performed using the Cox proportional hazards regression model. Hazard ratios (HRs) with 95% confidence intervals (CIs) were calculated as estimates of the correlations. p-values less than 0.05 were considered statistically significant.

## Results

### Clinicopathologic characteristics

A total of 598 surgically resected FFPE primary gastric cancer samples in TMA were assessed for FOXP3, CD163, PD-L1, CD3, and CD8 markers, including 418 males and 180 females (Additional file 1: Fig. S1). The median overall survival time is 48.1 months (range: 0–123 months), and the median age at diagnosis is 62 years (range: 26–88 years). The tissue samples consist of 14 cases of papillary adenocarcinoma (pap), 183 cases of tubular adenocarcinoma (tub), 316 cases of poor-differentiated adenocarcinoma (por), 22 cases of mucinous adenocarcinoma (muc) and 63 cases of signet ring cell (sig) type (pathological classification by Japanese classification). In total, 124 GC lesions occurred in “the upper parts of the stomach”, 197 GC tumors occurred in “the middle parts of the stomach”, and 277 GC cases occurred in “the lower parts of the stomach” [6].

The tumors were classified based on clinical TNM stages, including stage 1 (18.1%), stage 2 (25.1%), stage 3 (44.5%), and stage 4 (12.3%). The 1-, 3-, and 5-year overall survival rates were 1.00, 0.991 ± 0.009, and 0.954 ± 0.023, respectively, for stage 1; 0.927 ± 0.021, 0.827 ± 0.031, and 0.751 ± 0.036, respectively, for stage 2; the rates were 0.838 ± 0.023, 0.444 ± 0.030, and 0.363 ± 0.030, respectively, for stage 3; and the rates were 0.689 ± 0.054,0.132 ± 0.040, and 0.053 ± 0.028, respectively, for stage 4 (Table 1).

**Table 1:** Clinicopathologic characteristics of the total 598 gastric cancer patients

	n	T cells									M2 macrophages			Tumor cells		
		FOXP3			CD3			CD8			CD163			PD-L1		
		Low (%)	High (%)	*p* value	Low (%)	High (%)	p-value	Low (%)	High (%)	p-value	Low (%)	High (%)	p-value	Neg (%)	Pos (%)	p-value
Gender	598			0.285			0.858			0.285			0.373			0.43
Female	180	53.3	46.7		50.6	49.4		46.7	53.3		57.8	47.2		71.7	28.3	
Male	418	48.6	51.4		49.8	50.2		51.4	48.6		48.8	51.2		68.4	31.6	
Age	598			0.024			0.019			0.676			0.08			0.204
≤ 60	239	55.6	44.4		43.9	56.1		49	51		54.4	45.6		66.1	33.9	
> 60	359	46.2	53.8		53.8	46.2		50.7	49.3		47.1	52.9		71.6	28.4	
Location	598			0.334			0.473			0.321			0.186			0.977
U	124	55.6	44.4		54.8	45.2		53.2	46.8		53.2	46.8		70.2	29.8	
M	197	49.7	50.3		49.2	50.8		45.7	54.3		44.7	55.3		69	31	
L	277	47.7	52.3		48.4	51.6		51.6	48.4		52.3	47.7		69.3	30.7	
Pathological classification	598			< 0.001			< 0.001			< 0.001			0.004			< 0.001
Pap	14	28.6	71.4		85.7	14.3		92.9	7.1		50	50		64.3	35.7	
Tub	183	49.2	50.8		54.1	45.9		53.6	46.4		51.9	48.1		51.4	48.6	
Por	316	48.4	51.6		42.7	57.3		44.6	55.4		43.7	56.3		78.8	21.2	
Sig & Muc	85	61.2	38.8		62.4	37.6		55.3	44.7		68.2	31.8		75.3	24.7	
T stage	598			0.097			0.044			0.002			0.632			< 0.001
1	68	52.9	47.1		51.5	48.5		57.4	42.6		51.5	48.5		48.5	51.5	
2	84	52.4	47.6		41.7	58.3		45.2	54.8		45.2	54.8		58.3	41.7	
3	141	57.4	42.6		43.3	56.7		37.6	62.4		47.5	52.5		66	34	
4	35	45.2	54.8		55.1	44.9		55	45		52.1	47.9		78.7	21.3	
N stage	598			0.176			0.499			0.398			0.612			< 0.001
N0	224	46.4	53.6		48.2	51.8		47.8	52.2		51.3	48.7		53.6	46.4	
N+	374	52.1	47.9		51.1	48.9		51.3	48.7		49.2	50.8		78.9	21.1	
M stage	598			< 0.001			0.013			< 0.001			0.001			0.002
M0	524	55.7	44.3		48.1	51.9		46.6	53.4		52.7	47.3		67.2	32.8	
M+	74	9.5	90.5		63.5	36.5		74.3	25.7		31.1	68.9		85.1	14.9	
pTNM	598			< 0.001			0.082			< 0.001			0.001			< 0.001
1	108	49.1	50.9		47.2	52.8		54.7	45.3		51	49		47.3	52.7	
2	150	57.3	42.7		46	54		40	60		58	42		50.7	49.3	
3	266	57.5	42.5		49.6	50.4		47	53		54.5	45.5		80.5	19.5	
4	74	9.5	90.5		63.5	36.5		74.3	25.7		31.1	68.9		85.1	14.9	

U: upper parts; M: middle parts; L: lower parts; N0: no lymph node metastasis; N+: lymph node metastasis; Pap: papillary adenocarcinoma; Tub: tubular adenocarcinoma; Por: poor-differentiated adenocarcinoma; Sig: signet ring cell adenocarcinoma; Muc: mucinous adenocarcinoma; Neg: Negative; Pos: Postive

Regarding all 598 GC patients, univariate survival analysis revealed that age, pathological classification, T stage, N stage, M stage, pathological TNM stage, PD-L1 expression, and CD3, CD8, and CD163 infiltration were all significantly associated with patients prognosis (Additional file 2: Table S1). A strong negative correlation was noted between patients’ prognosis and the pathological TNM stages. GC patients with positive PD-L1 expression in tumor cells exhibited better survival (5-year OS, 68.4 ± 0.035 vs. 46.3 ± 0.025, p < 0.001). Moreover, CD3 and CD8 also exhibited the same tendency (5-year OS for CD3, 57.3 ± 0.029 vs 48.9 ± 0.030, p = 0.049; 5-year OS for CD8, 58.3 ± 0.029 vs 48.3 ± 0.030, p = 0.009). Nevertheless, CD163 exhibits a contrary outcome (5-year OS, 49.3 ± 0.030 vs. 80.3 ± 0.023, p = 0.012) (Additional files 3, 4, 5: Tables S2, S3, S4).

At the same time, in the multivariable survival analysis which includes age, pathological classification, T stage, N stage, M stage, PD-L1 expression, and CD163^+^M2 macrophage, CD3^+^ T lymphocyte, and CD8^+^ T lymphocyte cell density showed that T stage, N stage, M stage and CD163^+^M2 macrophages are independent significant poor prognostic factors. However, CD8^+^T lymphocytes are a favorable prognostic factor (Additional file 2: Table S1).

### Correlation of FOXP3^+^ Tregs and CD163^+^M2 macrophages with GC patients’ clinicopathological features

In our research, the correlation between FOXP3^+^Tregs and patients’ prognosis features did not show significant differences among the 598 samples (5-year OS, 51.4 ± 0.029 vs. 55.0 ± 0.029, p = 0.108). However, interestingly, when patients were divided into the earlier stage (stages I–II) and the later stage (stages III–IV), high FOXP3 expression indicates better prognosis (5-year OS, 90.0 ± 0.024 vs. 74.9 ± 0.037 p < 0.001) in the earlier stage group, and FOXP3^+^ Treg could be an independent favorable prognostic factor in the earlier stage subgroup (HR: 0.207, 95% CI 0.100–0.426, p < 0.001) (Table 2).

**Table 2:** Univariable and multivariable analysis of total and subgroups of gastric cancer patients

	Univariable				Multivaraible			
	p-value	HR	95% CI		p-value	HR	95% CI	
Total								
CD163 High vs Low	0.014	1.334	1.059	1.273	0.024*	1.335	1.04	1.715
PD-L1 Pos vs Neg	< 0.001	0.521	0.396	0.687	0.087	0.775	0.579	1.038
CD3 High vs Low	0.031	0.776	0.616	0.978	0.456	0.906	0.698	1.175
CD8 High vs Low	0.008	0.731	0.58	0.921	0.037*	0.75	0.573	0.982
FOXP3^low^CD163^low^	0.012	0.719	0.557	0.929				
Stage I–II								
FOXP3 High vs Low	< 0.001	0.231	0.112	0.474	< 0.001*	0.207	0.100	0.426
Stage III–IV								
CD8 High vs Low	0.005	0.694	0.537	0.897	0.003*	0.661	0.502	0.871
FOXP3^low^CD163^low^	< 0.001	0.547	0.408	0.734	< 0.001*	0.561	0.407	0.774
N+								
PD-L1 Pos vs Neg	0.001	0.535	0.373	0.768	0.011*	0.621	0.431	0.895
CD3 High vs Low	0.017	0.728	0.561	0.946	0.333	0.864	0.643	1.161
CD8 High vs Low	0.004	0.679	0.523	0.883	0.035*	0.719	0.53	0.977
FOXP3^low^CD163^low^	0.003	0.651	0.488	0.868	0.007*	0.654	0.479	0.892
Upper								
FOXP3^high^PD-L1^neg^	< 0.001	2.623	1.666	4.13	0.002*	2.123	1.309	3.443
Middle								
PD-L1 Pos vs Neg	0.005	0.443	2.252		0.075	0.594	0.336	1.053
CD8 High vs Low	0.029	0.606	0.387		0.015*	0.567	0.358	0.896
Lower								
PD-L1 Pos vs Neg	0.003	0.55	0.37	0.819	0.522	0.869	0.566	1.334
CD8 High vs Low	0.034	0.692	0.492	0.973	0.042*	0.692	0.485	0.987
Tub								
CD163 High vs Low	0.040	1.647	1.022	2.653	0.026*	1.765	1.071	2.908
PD-L1 Pos vs Neg	0.002	0.462	0.281	0.759				
FOXP3^high^PD-L1^neg^	0.014	1.868	1.137	3.070	0.227	1.387	0.815	2.316
Por								
FOXP3 High vs Low	0.018	1.442	1.065	1.951	0.949	0.988	0.691	1.414
CD163 High vs Low	0.015	1.462	1.075	1.989	0.277	1.215	0.855	1.724
CD3 High vs Low	0.002	0.617	0.458	0.832	0.246	0.814	0.574	1.153
CD8 High vs Low	0.01	0.677	0.502	0.913	0.499	0.883	0.617	1.266
Sig+Muc								
FOXP3^high^CD163^high^	0.029	0.206	0.05	0.852	0.011*	0.154	0.036	0.656
“a+b+c”								
FOXP3^low^CD163^low^	0.001	0.319	0.161	0.634	0.001*	0.319	0.161	0.634
“a+b”								
FOXP3 High vs Low	0.006	0.502	0.308	0.818	0.022*	0.563	0.344	0.92
CD8 High vs Low	0.003	0.506	0.323	0.793	0.006*	0.533	0.34	0.837

HR hazard ratio, CI confidence interval; N+: with lymph node metastasis; Pap: papillary adenocarcinoma; Tub: tubular adenocarcinoma; Por: poor-differentiated adenocarcinoma; Sig: signet ring cell addenocaecinoma; Muc: mucinous adenocarcinoma; “a+b+c”: chemotherapy with fluorouracil cisplatin and paclitaxel; “a+b”: chemotherapy with fluorouracil and cisplatin

* p-value < 0.05 in multivariable analysis

**Fig. 1:**
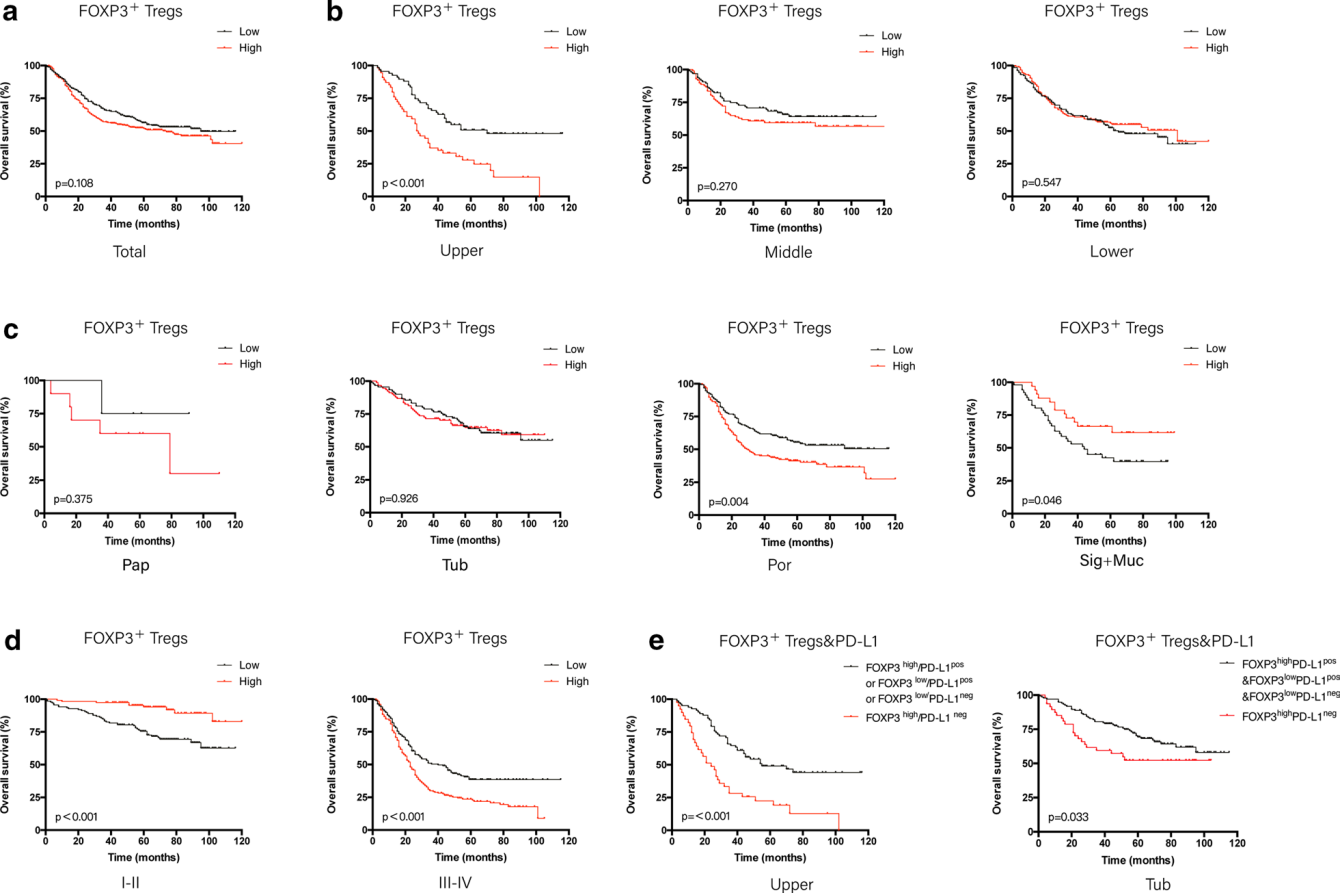
Correlation of FOXP3^+^Tregs with GC patients’ overall survival. Kaplan–Meier survival curves for OS based on FOXP3 in total GC patients (**a**); in different locations (**b**); in different pathological classifications (**c**); in different TNM stages (**d**); and based on FOXP3 combined with PD-L1 in certain subgroups of GC (**e**). Pap: papillary adenocarcinoma; Tub: tubular adenocarcinoma; Por: poor differentiated adenocarcinoma; Sig: signet ring cell adenocarcinoma; Muc: mucinous adenocarcinoma

**Fig. 2:**
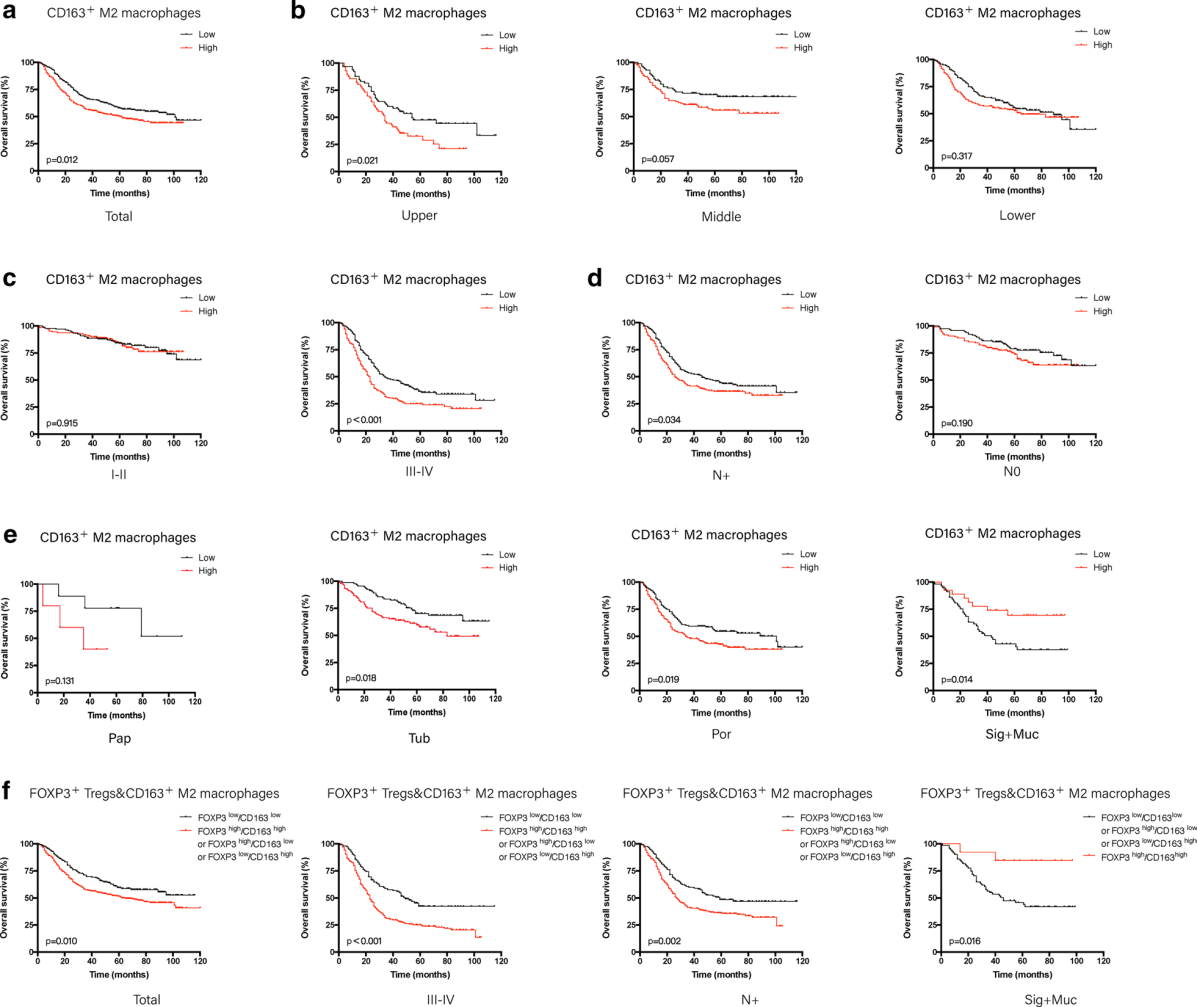
Correlation of CD163^+^M2 macrophages with GC patients’ overall survival. Kaplan–Meier survival curves for OS based on CD163 in total GC patients (**a**); in different locations (**b**); in different TNM stages (**c**); with or without lymph node metastasis (**d**); in different pathological classifications (**e**); and based on CD163 combined with FOXP3 in total and certain subgroups of GC (**f**)

Nevertheless, in the later stage group, the high expression of FOXP3 indicates poor prognosis (5-year OS, 23.3 ± 0.032 vs. 37.1 ± 0.040 p < 0.001). This phenomenon also indicates that FOXP3^+^ Treg cells may play a diverse role in the progression of GC (Fig. 1). CD163^+^M2 macrophages could be regarded as an independent poor prognostic factor in all samples (HR: 1.335, 95% CI 1.040–1.715, p = 0.024). M2 macrophages indicated poor prognosis in general. However, high M2 macrophage infiltration suggests a favorable prognosis in signet ring cell carcinoma and mucinous adenocarcinoma (5-year OS, 49.3 ± 0.030 vs 80.3 ± 0.023, p = 0.012) (Additional file 6: Table S5, Fig. 2e).

### Correlation of CD8^+^ lymphocytes, CD3^+^ lymphocytes and PD-L1 with GC patients’ clinicopathological features

CD8^+^ T lymphocytes could be independent favorable prognostic factor in all samples (HR: 0.750, 95% CI 0.573–0.982, p = 0.037). High CD8 expression indicates better prognosis (5-year OS, 58.3 ± 0.029 vs 48.3 ± 0.030, p = 0.009). It could also be an independent favorable prognostic factor in certain subgroups of GC. Nevertheless, CD3^+^T lymphocytes are not an independent favorable prognostic factor in the total sample population or any subgroup of GC (Additional files 7, 8: Figs. S3, S4; Table 2). In this study, 183 out of 598 cases (30.6%) showed positive PD-L1 expression in tumor cells, whereas 415 patients out of 598 cases (69.4%) showed negative PD-L1 expression in tumor cells. In general, the positive expression of PD-L1 indicates a better prognosis. Positive PD-L1 expression could be an independent favorable prognostic factor in the lymph node metastasis subgroup (HR: 0.621, 95% CI 0.431–0.895, p = 0.011) (Additional file 9: Fig. S2, Table 2).

### Prognostic value of the combination of FOXP3^+^Tregs, CD163^+^M2 macrophages, and PD-L1 in GC patients

Using two-category immunoscore analysis, combined FOXP3 and CD163, FOXP3^low^CD163^low^, showed better prognosis in the later subgroup (stage III-IV) (5-year OS, 24.5 ± 0.029 vs. 41.1 ± 0.051, p < 0.001). FOXP3^low^CD163^low^ is an independent favorable prognostic factor in this subgroup (HR: 0.561, 95% CI 0.407–0.774, p < 0.001) and is also an independent favorable prognostic factor in the lymph node metastasis subgroup (HR: 0.654, 95% CI 0.479–0.892, p = 0.007). Nevertheless, FOXP3^high^ CD163^high^ is an independent favorable prognostic factor in the signet ring cell and mucinous adenocarcinoma subgroup (HR: 0.154, 95% CI 0.036–0.656, p = 0.011) (Fig. 2f). Then, when FOXP3 was combined with PD-L1, FOXP3^high^ PD-L1^neg^ could be regarded as a poor prognostic factor in the upper parts subgroup (HR: 2.123, 95% CI 1.309–3.443, p = 0.002) (Fig. 1e; Table 2).

### Immune indicators predict different prognosis in stage II-III GC patients treated with different chemotherapy strategies

In this section, we focused on GC patients who are eligible for radical resection. Stage I GC patients did not receive chemotherapy, and stage IV GC patients had distant metastases and were unable to undergo radical resection [34]. Thus, stage II-III GC patients were mainly discussed. First-line chemotherapy for GC is platinum and fluorouracil. When the patient relapses, the second-line chemotherapy regimen paclitaxel is added. Thus, the two main chemotherapy strategies are fluorouracil, cisplatin and paclitaxel (a+b+c) and fluorouracil and cisplatin (a+b).

FOXP3^low^CD163^low^ was an independent favorable prognostic factor in the (a+b+c) group.(HR: 0.319, 95% CI 0.161–0.634, p = 0.001). However, in the (a+b) group, N stage is an independent poor prognostic factor. (HR: 2.384, 95% CI 1.379–4.121, p = 0.002). Both FOXP3 and CD8 are independent favorable prognostic factors (HR: 0.563, 95% CI 0.344–0.920, p = 0.022; HR: 0.533, 95% CI 0.340–0.837, p = 0.006, respectively) (Additional file 10: Fig. S5, Additional file 11: Table S6).

## Discussion

Immune cells in the tumor microenvironment have a crucial influence on tumor occurrence and development [35]. In this study, we conducted an immunohistochemical evaluation of specific immune indices from 598 GC samples. We mainly focused on specific immune cells and immune indicators that exhibit different prognostic value in different subgroups of GC patients.

In this research, CD3^+^ T lymphocytes, CD8^+^ T lymphocytes, and PD-L1 are generally favorable prognostic indicators. Many studies have exhibited that a large number of Tregs infiltrate into various types of tumors in humans. A high frequency of tumor-infiltrating FOXP3^+^ Tregs was often significantly negatively correlated with patient survival [36, 37]. To date, the function of FOXP3^+^Treg in GC patients and the evaluation of prognosis have been controversial. Some studies have explained that high expression of Tregs in GC suggests poor prognosis, which is related to lymph node metastasis, late stage, and poor pathological types. Some studies imply that high Treg infiltration is a good predictor of prognosis. However, these studies do not distinguish the pathological stages of GC, which is only discussed in general terms [22, 23]. In this study, the GC patients were first divided into earlier and later stages for discussion. In earlier stage GC patients, there was a tendency of high FOXP3^+^Treg infiltration, indicating a good prognosis. However, in lated stage GC patients, the reverse trend was noted. In recent years, many studies have also discussed the different functions of various subsets of tumor-infiltrating Tregs [38–40]. These findings indicate that more attention to the basic research of FOXP3+ Treg cells should be paid, and different functions of FOXP3+ Treg cells in different stages of GC may be explored. The disparate prognostic implications of FOXP3^+^Tregs in GC were based on different pathological classifications and different locations,which further illustrate the complexity of FOXP3^+^Treg’s function in different GC subgroups.

Regarding the infiltrating status of various immune cells in different subgroups of GC, FOXP3Tregs showed the lowest level in signet ring cell carcinoma and mucinous adenocarcinoma but higher in the lower parts subgroup, compared with the middle and upper parts subgroups. The levels of CD163^+^ M2 macrophage were low in signet ring cell carcinoma and mucinous adenocarcinoma but ample in poor-differentiated adenocarcinoma (Additional file 12: Fig. S6). M2 macrophage is a factor that could promote tumor progression in many tumors. A meta-analysis showed that the number of infiltrating M2 macrophages and total TAMs might be negative prognostic factors for GC patients, while M1 macrophage infiltration may be associated with a favorable survival rate [41]. However, in some specific subgroups of GC, different prognostic implications of M2 macrophages for patients were rarely reported. This study observed that M2 macrophages might be highly infiltrated in signet ring cell carcinoma and mucinous adenocarcinoma, which suggests a good prognosis. However, more specimens and basic studies are required to further support this result.

In this research, the positive expression of PD-L1 indicates better prognosis, which is consistent with the tendency reported [29]. However, discrepancies in the percentage of the PD-L1 positive expression patients were ascribing to the variation in the cut-off values, which still exhibit significant heterogeneity [42]. The prognostic value of PD-L1 levels in GC patients with high CD8/FOXP3 and low CD8/PD-L1 were also reported [43], which enlightens the strategy of combining some indicators to predict GC patients’s prognosis. When combined FOXP3 with CD163 or PD-L1 to predict the prognosis of patients with different subtypes of GC, some combined prognostic factors become independent in multivariable subgroup analyses, which was of great significance. These findings could shed light on future GC immunotherapy which is not exclusively limited to block immune checkpoints. Furthermore, attention should be paid to the combined treatment of some specific Treg subsets and M2 macrophages.

Also, some immune indices indicate different prognostic value in stage II-III GC patients who were treated with different chemotherapy strategies. These results suggest the necessity of conducting basic research on some immune cells in different subgroups, such as different clinical, pathological stages, different chemotherapy regimens, different pathological types of GC, and direct further understanding about the heterogeneity and the immune status of GC.

In this study, large-scale GC samples were used to elucidate both CD3^+^, CD8^+^ T lymphocytes with positive prognostic effects. These studies included FOXP3^+^ Tregs and M2 macrophages given their different prognostic implications. Due to the limitations of single-center retrospective studies, more multicenter studies should be conducted to validate all of these results.

## Conclusions

Analysis of different subtypes of GC patients and identification of several immune indicators for different indications of prognosis in various subtypes of GC patients are the highlights of this study. This information would provide a further theoretical basis and a potential target for GC immunotherapy.

## Additional files

**Additional file 1: Fig. S1.** FOXP3, CD163, PD-L1, CD3 and CD8 expression in gastric cancer by immunohistochemistry (×200). The representative images of FOXP3+Tregs (a) CD163+M2 macrophages (b); the representative positive expression of PD-L1 in tumor tissues (c); the representative negative expression of PD-L1 in tumor tissues (d); CD3+T lympmcytes (e); CD8+T lympmcytes (f). (The lower panel :×400).

**Additional file 2: Table S1.** Univariable and multivariable analysis of total 598 gastric cancers.

**Additional file 3: Table S2.** Univariable and multivariable analysis in different stages of gastric cancers.

**Additional file 4: Table S3.** Univariable and multivariable analysis in gastric cancer with or without lymph node metastasis.

**Additional file 5: Table S4.** Univariable and multivariable analysis in different locations of gastric cancers.

**Additional file 6: Table S5.** Univariable and multivariable analysis in different pathological classifications of gastric cancers.

**Additional file 7: Fig. S3.** Correlation of CD3^+^T lymphcytes with GC patients’ overall survival. Kaplan-Meier survival curves for OS based on CD3 in total GC patients (a); in different locations (b); in different TNM stages (c); with or without lymph node metastasis (d); in different pathological classifications (e).

**Additional file 8: Fig. S4.** Correlation of CD8+T lymphcytes with GC patients’ overall survival. Kaplan-Meier survival curves for OS based on CD8 in total GC patients (a); in different locations (b); in different TNM stages (c); with or without lymph node metastasis (d); in different pathological classifications (e).

**Additional file 9: Fig. S2.** Correlation of PD-L1 with GC patients’ overall survival. Kaplan-Meier survival curves for OS based on PD-L1 in total GC patients (a); in different locations (b); in different TNM stages (c); with or without lymph node metastasis (d); in different pathological classifications (e).

**Additional file 10: Fig. S5.** Overall survival analysis in stage II-III GC patients treated with different chemotherapy strategies. (a): Kaplan-Meier survival curves for OS based on 5 immune indicators in stage II–III GC patients treated with chemotherapy strategy fluorouracil ,cisplatin and paclitaxel (a+b+c); (b): Kaplan-Meier survival curves for OS based on 5 immune indicators in stage II-III GC patients treated with chemotherapy strategy fluorouracil ,cisplatin (a+b).

**Additional file 11: Table S6.** Univariable and multivariable analysis in different chemotherapy strategies of II–III gastric cancer.

**Additional file 12: Fig. S6.** Cell counts comparison in different subgroups of GC. Cell counts comparison in different TNM stages of GC (a); with or without lymph node metastasis (b); in different pathological classifications (c); in different locations of GC (d); in different chemotherapy strategies of stage II–III GC (e). (*p < 0.05; **p < 0.01; ***p < 0.001;****p < 0.0001; ns: no statistical significance).

## Data Availability

The datasets used and/or analysed during the current study are available from the corresponding author on reasonable request.

## Abbreviations

CI: confidence interval
FFPE: formalin-fixed, paraffin-embedded
GC: gastric cancer
HR: hazard ratio
LN: lymph node
OS: overall survival
Treg: regulatory T cell
TAMs: tumor-associated macrophages
TMA: tissue microarray
TILs: tumor-infiltrating lymphocytes
por: poor-differentiated adenocarcinoma
pap: papillary adenocarcinoma
tub: tubular adenocarcinoma
muc: mucinous adenocarcinoma
sig: signet ring cell carcinoma
