# Supplementary figures and images for: Regulatory T cells and M2 macrophages present diverse prognostic value in gastric cancer patients with different clinicopathologic characteristics and chemotherapy strategies
Source: J Transl Med. 2019 Jun 7;17:192. doi: 10.1186/s12967-019-1929-9 (PMC6554965; doi:10.1186/s12967-019-1929-9)

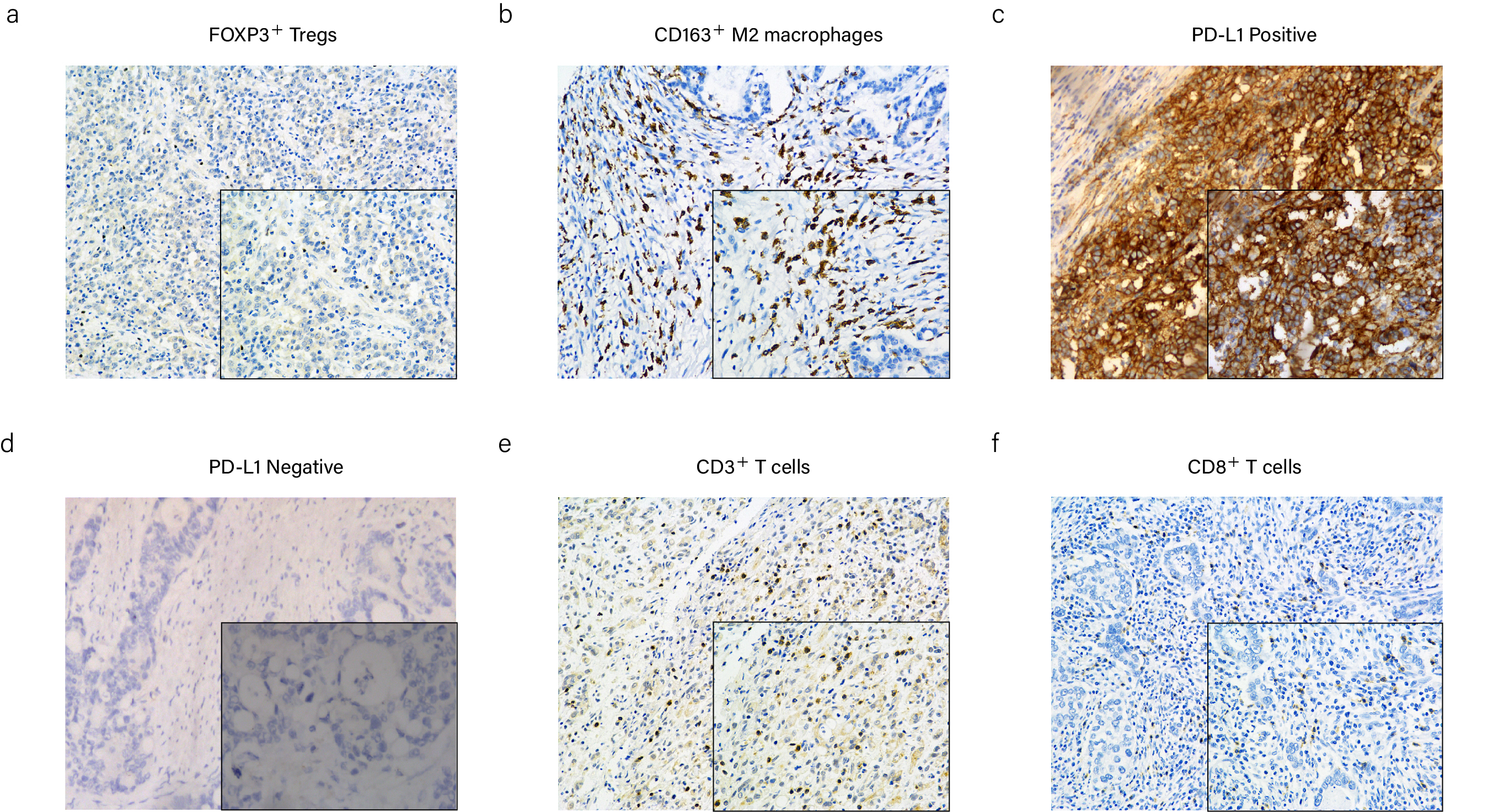

Supplement: Supplementary file 1 — Additional file 1: Fig. S1. FOXP3, CD163, PD-L1, CD3 and CD8 expression in gastric cancer by immunohistochemistry (×200). The representative images of FOXP3+Tregs (a) CD163+M2 macrophages (b); the representative positive expression of PD-L1 in tumor tissues (c); the representative negative expression of PD-L1 in tumor tissues (d); CD3+T lympmcytes (e); CD8+T lympmcytes (f). (The lower panel :×400). [file 12967_2019_1929_MOESM1_ESM.tif]

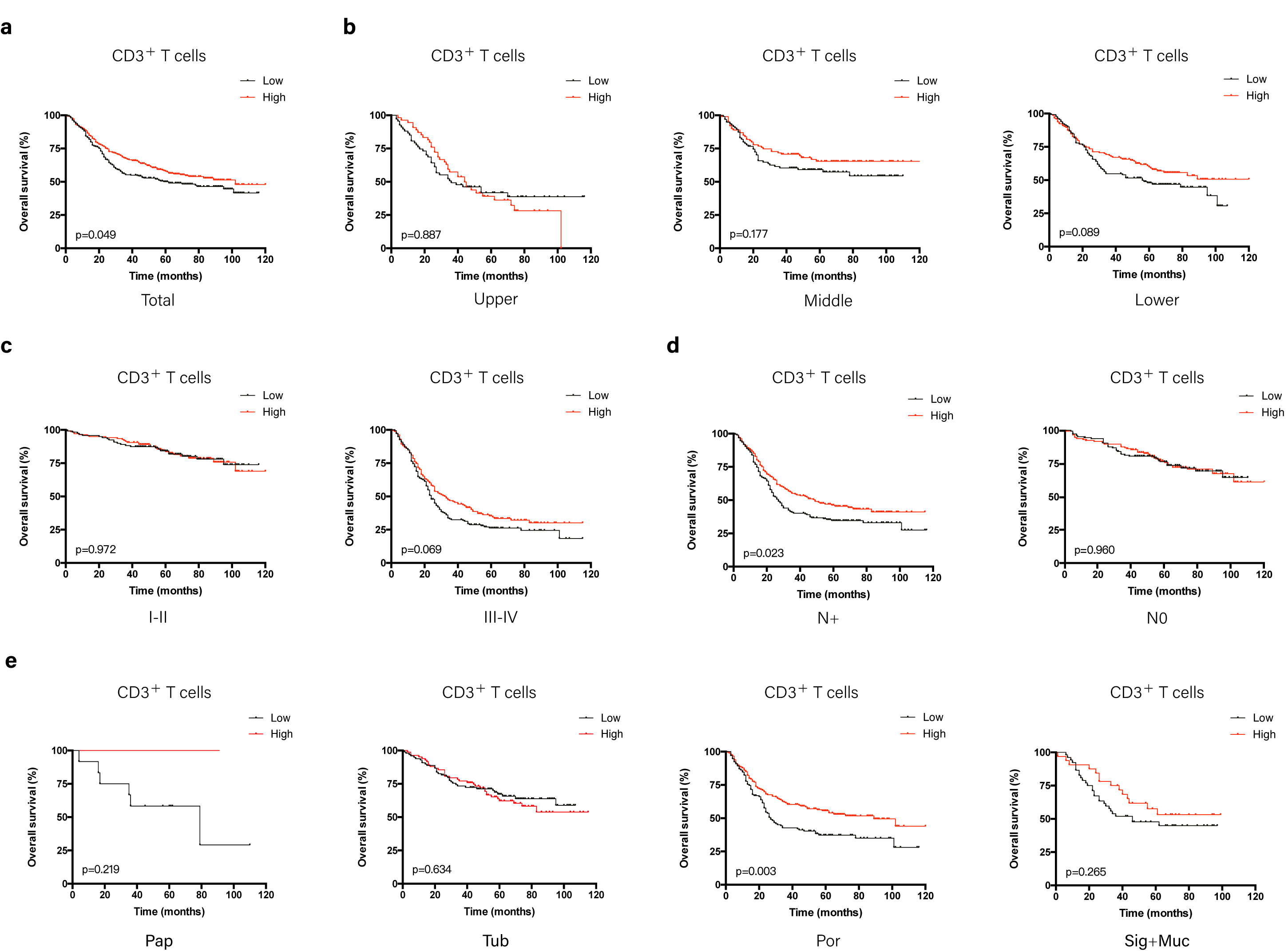

Supplement: Supplementary file 7 — Additional file 7: Fig. S3. Correlation of CD3+T lymphcytes with GC patients’ overall survival. Kaplan-Meier survival curves for OS based on CD3 in total GC patients (a); in different locations (b); in different TNM stages (c); with or without lymph node metastasis (d); in different pathological classifications (e). [file 12967_2019_1929_MOESM7_ESM.tif]

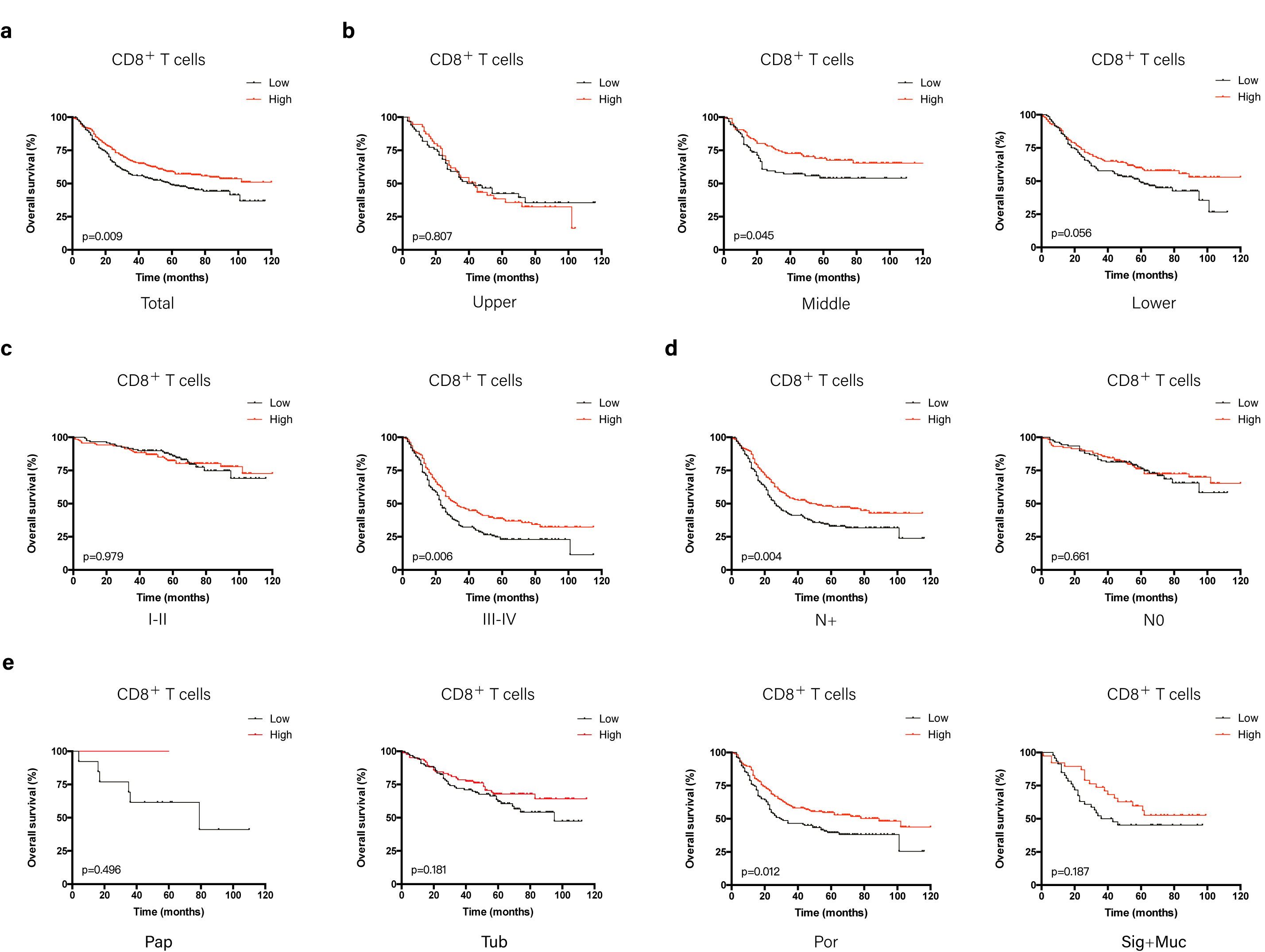

Supplement: Supplementary file 8 — Additional file 8: Fig. S4. Correlation of CD8+T lymphcytes with GC patients’ overall survival. Kaplan-Meier survival curves for OS based on CD8 in total GC patients (a); in different locations (b); in different TNM stages (c); with or without lymph node metastasis (d); in different pathological classifications (e). [file 12967_2019_1929_MOESM8_ESM.tif]

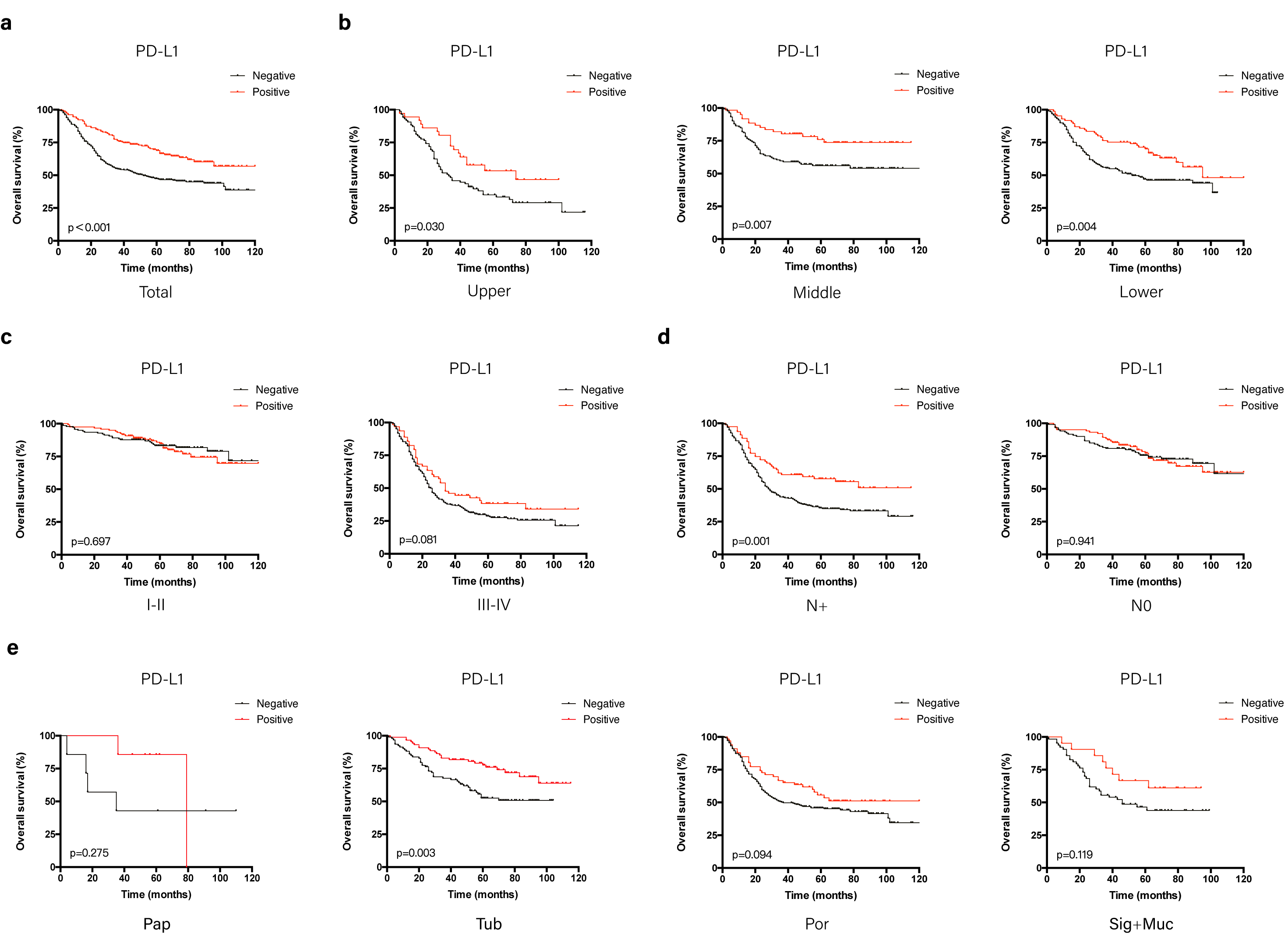

Supplement: Supplementary file 9 — Additional file 9: Fig. S2. Correlation of PD-L1 with GC patients’ overall survival. Kaplan-Meier survival curves for OS based on PD-L1 in total GC patients (a); in different locations (b); in different TNM stages (c); with or without lymph node metastasis (d); in different pathological classifications (e). [file 12967_2019_1929_MOESM9_ESM.tif]

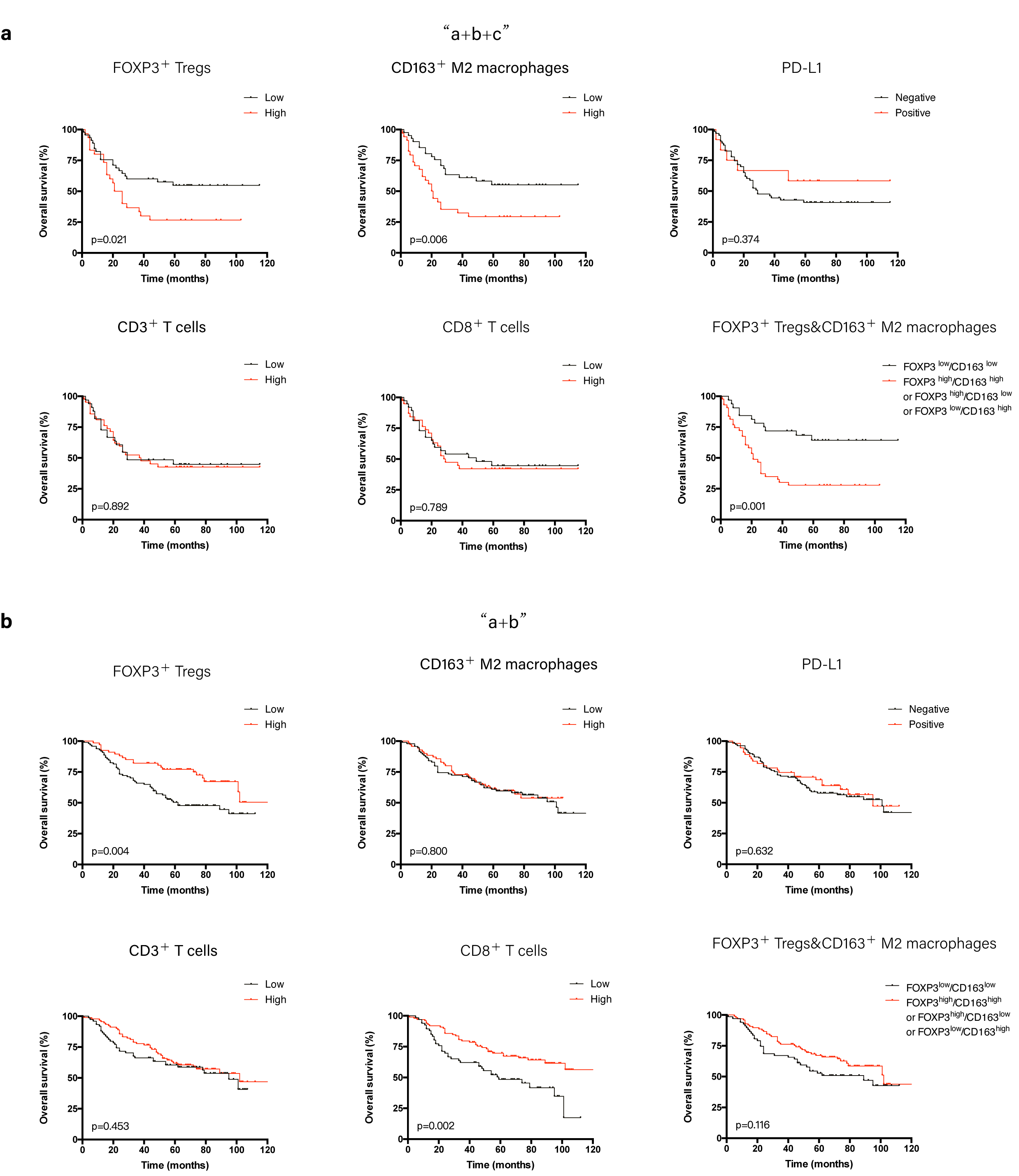

Supplement: Supplementary file 10 — Additional file 10: Fig. S5. Overall survival analysis in stage II-III GC patients treated with different chemotherapy strategies. (a): Kaplan-Meier survival curves for OS based on 5 immune indicators in stage II–III GC patients treated with chemotherapy strategy fluorouracil ,cisplatin and paclitaxel (a+b+c); (b): Kaplan-Meier survival curves for OS based on 5 immune indicators in stage II-III GC patients treated with chemotherapy strategy fluorouracil ,cisplatin (a+b). [file 12967_2019_1929_MOESM10_ESM.tif]

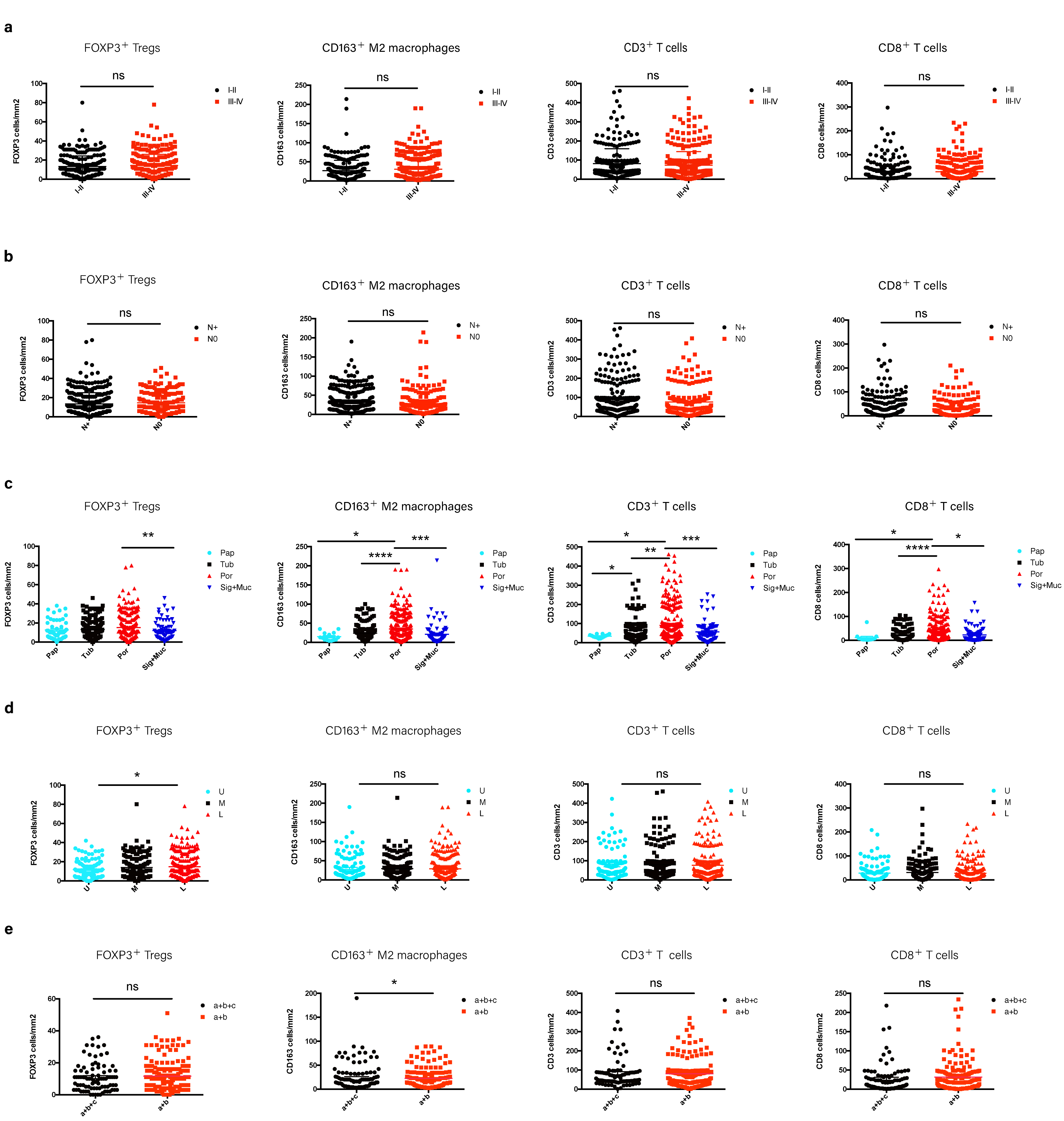

Supplement: Supplementary file 12 — Additional file 12: Fig. S6. Cell counts comparison in different subgroups of GC. Cell counts comparison in different TNM stages of GC (a); with or without lymph node metastasis (b); in different pathological classifications (c); in different locations of GC (d); in different chemotherapy strategies of stage II–III GC (e). (*p < 0.05; **p < 0.01; ***p < 0.001;****p < 0.0001; ns: no statistical significance). [file 12967_2019_1929_MOESM12_ESM.tif]
