# Supplementary material for: Regulatory T cells and M2 macrophages present diverse prognostic value in gastric cancer patients with different clinicopathologic characteristics and chemotherapy strategies
Source: J Transl Med. 2019 Jun 7;17:192. doi: 10.1186/s12967-019-1929-9 (PMC6554965; doi:10.1186/s12967-019-1929-9)
Supplement: Supplementary file 2 — Additional file 2: Table S1. Univariable and multivariable analysis of total 598 gastric cancers. [file 12967_2019_1929_MOESM2_ESM.docx]

| **Table S1.Univariable and multivariable analysis of total 598 gastric cancers** | | | | | | | | |
| --- | --- | --- | --- | --- | --- | --- | --- | --- |
|  | **Univariable** | | | | **Multivaraible** | | | |
|  | p-value | HR | 95% CI | | p-value | HR | 95% CI | |
| Age | 0.003 | 1.017 | 1.006 | 1.029 | 0.197 | 1.009 | 0.996 | 1.022 |
| Gender | 0.509 | 1.087 | 0.848 | 1.393 |  |  |  |  |
| Location | 0.089 | 0.88 | 0.76 | 1.02 |  |  |  |  |
| Pathological classification | 0.001 | 1.129 | 1.084 | 1.372 | 0.126 | 1.102 | 0.973 | 1.249 |
| T stage |  |  |  |  |  |  |  |  |
| 1 | ＜0.001 |  |  |  | ＜0.001 |  |  |  |
| 2 | ＜0.001 | 0.181 | 0.086 | 0.383 | ＜0.001 | 0.252 | 0.118 | 0.536 |
| 3 | 0.42 | 0.845 | 0.561 | 1.273 | 0.743 | 0.933 | 0.618 | 1.409 |
| 4 | ＜0.001 | 2.188 | 1.582 | 3.025 | ＜0.001 | 2.005 | 1.432 | 2.809 |
| N stage | ＜0.001 | 3.161 | 2.384 | 4.191 | ＜0.001 | 1.991 | 1.485 | 2.67 |
| M stage | ＜0.001 | 4.452 | 3.355 | 5.908 | ＜0.001 | 2.722 | 1.972 | 3.757 |
| pTNM |  |  |  |  |  |  |  |  |
| 1 | ＜0.001 |  |  |  |  |  |  |  |
| 2 | ＜0.001 | 5.145 | 2.317 | 11.423 |  |  |  |  |
| 3 | ＜0.001 | 16.885 | 7.921 | 35.993 |  |  |  |  |
| 4 | ＜0.001 | 41.263 | 18.817 | 91.481 |  |  |  |  |
| FOXP3 High vs Low | 0.183 | 1.169 | 0.929 | 1.472 |  |  |  |  |
| CD163 High vs Low | 0.014 | 1.334 | 1.059 | 1.273 | 0.024 | 1.335 | 1.04 | 1.715 |
| PD-L1 Pos vs Neg | ＜0.001 | 0.521 | 0.396 | 0.687 | 0.087 | 0.775 | 0.579 | 1.038 |
| CD3 High vs Low | 0.031 | 0.776 | 0.616 | 0.978 | 0.456 | 0.906 | 0.698 | 1.175 |
| CD8 High vs Low | 0.008 | 0.731 | 0.58 | 0.921 | 0.037 | 0.75 | 0.573 | 0.982 |
| FOXP3^low^CD163^low^ | 0.012 | 0.719 | 0.557 | 0.929 |  |  |  |  |
| HR:hazard ratio | | CI:Confidence intervals | |  |  |  |  |  |
