# Supplementary material for: Regulatory T cells and M2 macrophages present diverse prognostic value in gastric cancer patients with different clinicopathologic characteristics and chemotherapy strategies
Source: J Transl Med. 2019 Jun 7;17:192. doi: 10.1186/s12967-019-1929-9 (PMC6554965; doi:10.1186/s12967-019-1929-9)
Supplement: Supplementary file 3 — Additional file 3: Table S2. Univariable and multivariable analysis in different stages of gastric cancers. [file 12967_2019_1929_MOESM3_ESM.docx]

| **Table S2.Univariable and multivariable analysis in different stages of gastric cancers** | | | | | | | | |
| --- | --- | --- | --- | --- | --- | --- | --- | --- |
|  | **Univariable** | | | | **Multivariable** | | | |
| Stage I-II |  |  |  | |  |  |  | |
|  | p-value | HR | 95%CI | | p-value | HR | 95%CI | |
| Age | 0.043 | 1.028 | 1.001 | 1.057 | 0.083 | 1.024 | 0.997 | 1.053 |
| Gender | 0.539 | 1.2 | 0.67 | 2.15 |  |  |  |  |
| Location | 0.602 | 1.117 | 0.737 | 1.695 |  |  |  |  |
| Pathological classification | 0.214 | 1.184 | 0.907 | 1.545 |  |  |  |  |
| T stage |  |  |  |  |  |  |  |  |
| 1 | 0.006 |  |  |  | 0.008 |  |  |  |
| 2 | 0.002 | 0.256 | 0.107 | 0.613 | 0.002 | 0.25 | 0.104 | 0.601 |
| 3 | 0.331 | 1.274 | 0.782 | 2.076 | 0.259 | 1.325 | 0.813 | 2.161 |
| 4 | 0.001 | 2.298 | 1.388 | 3.803 | 0.002 | 2.207 | 1.326 | 3.676 |
| N stage | 0.251 | 1.415 | 0.782 | 2.562 |  |  |  |  |
| FOXP3 High vs Low | ＜0.001 | 0.231 | 0.112 | 0.474 | ＜0.001 | 0.207 | 0.1 | 0.426 |
| CD163 High vs Low | 0.804 | 1.072 | 0.618 | 1.859 |  |  |  |  |
| PD-L1 Pos vs Neg | 0.594 | 1.161 | 0.671 | 2.011 |  |  |  |  |
| CD3 High vs Low | 0.908 | 0.968 | 0.558 | 1.678 |  |  |  |  |
| CD8 High vs Low | 0.917 | 1.03 | 0.593 | 1.79 |  |  |  |  |
|  |  |  |  |  |  |  |  |  |
| Stage III-IV |  |  |  |  |  |  |  |  |
| Age |  |  |  |  |  |  |  |  |
| Gender | 0.59 | 1.078 | 0.82 | 1.418 |  |  |  |  |
| Location | 0.712 | 1.029 | 0.884 | 1.197 |  |  |  |  |
| Pathological classification | 0.406 | 1.058 | 0.926 | 1.209 |  |  |  |  |
| T stage |  |  |  |  |  |  |  |  |
| 1 | 0.353 |  |  |  |  |  |  |  |
| 2 | 0.856 | 1.225 | 0.137 | 10.976 |  |  |  |  |
| 3 | 0.619 | 0.605 | 0.083 | 4.382 |  |  |  |  |
| 4 | 0.782 | 0.758 | 0.106 | 5.419 |  |  |  |  |
| N stage | 0.68 | 0.919 | 0.615 | 1.373 |  |  |  |  |
| M stage | ＜0.001 | 2.33 | 1.747 | 3.108 | ＜0.001 | 1.763 | 1.287 | 2.415 |
| FOXP3 High vs Low | 0.001 | 1.574 | 1.215 | 2.04 |  |  |  |  |
| CD163 High vs Low | 0.002 | 1.508 | 1.169 | 1.947 |  |  |  |  |
| PD-L1 Pos vs Neg | 0.051 | 0.711 | 0.504 | 1.002 |  |  |  |  |
| CD3 High vs Low | 0.055 | 0.779 | 0.604 | 1.005 |  |  |  |  |
| CD8 High vs Low | 0.005 | 0.694 | 0.537 | 0.897 | 0.003 | 0.661 | 0.502 | 0.871 |
| FOXP3^low^CD163^low^ | ＜0.001 | 0.547 | 0.408 | 0.734 | ＜0.001 | 0.561 | 0.407 | 0.774 |
