# Supplementary material for: Regulatory T cells and M2 macrophages present diverse prognostic value in gastric cancer patients with different clinicopathologic characteristics and chemotherapy strategies
Source: J Transl Med. 2019 Jun 7;17:192. doi: 10.1186/s12967-019-1929-9 (PMC6554965; doi:10.1186/s12967-019-1929-9)
Supplement: Supplementary file 4 — Additional file 4: Table S3. Univariable and multivariable analysis in gastric cancer with or without lymph node metastasis. [file 12967_2019_1929_MOESM4_ESM.docx]

| **Table S3.Univariable and multivariable analysis in gastric cancer with or without lymph node metastasis** | | | | | | | | |
| --- | --- | --- | --- | --- | --- | --- | --- | --- |
|  | **Univariable** | | | | **Multivariable** | | | |
|  |  |  |  | |  |  |  | |
|  | p-value | HR | 95%CI | | p-value | HR | 95%CI | |
| N0 |  |  |  | |  |  |  | |
| Age | 0.01 | 1.033 | 1.008 | 1.059 |  |  |  |  |
| Gender | 0.077 | 1.582 | 0.951 | 2.63 |  |  |  |  |
| Location | 0.174 | 0.79 | 0.562 | 1.11 |  |  |  |  |
| Pathological classification | 0.021 | 1.339 | 1.044 | 1.718 | 0.416 | 1.116 | 0.857 | 1.453 |
| T stage |  |  |  |  |  |  |  |  |
| 1 | ＜0.001 |  |  |  | 0.002 |  |  |  |
| 2 | 0.001 | 0.226 | 0.094 | 0.543 | 0.003 | 0.258 | 0.107 | 0.622 |
| 3 | 0.636 | 0.872 | 0.495 | 1.537 | 0.865 | 1.052 | 0.588 | 1.882 |
| 4 | 0.001 | 2.386 | 1.416 | 4.019 | ＜0.001 | 2.681 | 1.583 | 4.541 |
| M stage | ＜0.001 | 21.564 | 10.961 | 42.421 | ＜0.001 | 17.25 | 8.256 | 36.045 |
| pTNM |  |  |  |  |  |  |  |  |
| 1 | ＜0.001 |  |  |  |  |  |  |  |
| 2 | ＜0.001 | 5.897 | 2.447 | 14.214 |  |  |  |  |
| 3 | ＜0.001 | 17.166 | 5.832 | 50.53 |  |  |  |  |
| 4 | ＜0.001 | 89.7 | 32.431 | 248.099 |  |  |  |  |
| FOXP3 High vs Low | 0.998 | 0.226 | 0.094 | 0.543 |  |  |  |  |
| CD163 High vs Low | 0.156 | 1.437 | 0.871 | 2.372 |  |  |  |  |
| PD-L1 Pos vs Neg | 0.949 | 1.016 | 0.618 | 1.673 |  |  |  |  |
| CD3 High vs Low | 0.857 | 0.955 | 0.58 | 1.572 |  |  |  |  |
| CD8 High vs Low | 0.756 | 0.924 | 0.56 | 1.523 |  |  |  |  |
|  |  |  |  |  |  |  |  |  |
| N+ |  |  |  |  |  |  |  |  |
| Age | 0.174 | 1.009 | 0.996 | 1.023 |  |  |  |  |
| Gender | 0.965 | 0.994 | 0.746 | 1.323 |  |  |  |  |
| Location | 0.824 | 0.982 | 0.837 | 1.152 |  |  |  |  |
| Pathological classification | 0.109 | 1.116 | 0.976 | 1.275 |  |  |  |  |
| T stage |  |  |  |  |  |  |  |  |
| 1 | ＜0.001 |  |  |  | 0.004 |  |  |  |
| 2 | 0.058 | 0.238 | 0.054 | 1.047 | 0.079 | 0.264 | 0.06 | 1.164 |
| 3 | 0.69 | 0.874 | 0.449 | 1.699 | 0.643 | 0.854 | 0.438 | 1.665 |
| 4 | 0.04 | 1.783 | 1.027 | 3.095 | 0.015 | 1.981 | 1.139 | 3.443 |
| M stage | ＜0.001 | 2.825 | 2.045 | 3.903 | ＜0.001 | 1.924 | 1.342 | 2.757 |
| pTNM |  |  |  |  |  |  |  |  |
| 1 | ＜0.001 |  |  |  |  |  |  |  |
| 2 | 0.389 | 2.433 | 0.321 | 18.433 |  |  |  |  |
| 3 | 0.029 | 8.938 | 1.25 | 63.891 |  |  |  |  |
|  | 0.003 | 20.316 | 2.796 | 147.646 |  |  |  |  |
| FOXP3 High vs Low | 0.032 | 1.33 | 1.025 | 1.724 |  |  |  |  |
| CD163 High vs Low | 0.048 | 1.3 | 1.002 | 1.686 |  |  |  |  |
| PD-L1 Pos vs Neg | 0.001 | 0.535 | 0.373 | 0.768 | 0.011 | 0.621 | 0.431 | 0.895 |
| CD3 High vs Low | 0.017 | 0.728 | 0.561 | 0.946 | 0.333 | 0.864 | 0.643 | 1.161 |
| CD8 High vs Low | 0.004 | 0.679 | 0.523 | 0.883 | 0.035 | 0.719 | 0.53 | 0.977 |
| FOXP3^low^CD163^low^ | 0.003 | 0.651 | 0.488 | 0.868 | 0.007 | 0.654 | 0.479 | 0.892 |

N0:without lymph node metastasis; N+:with lymph node metastasis
