# Supplementary material for: Regulatory T cells and M2 macrophages present diverse prognostic value in gastric cancer patients with different clinicopathologic characteristics and chemotherapy strategies
Source: J Transl Med. 2019 Jun 7;17:192. doi: 10.1186/s12967-019-1929-9 (PMC6554965; doi:10.1186/s12967-019-1929-9)
Supplement: Supplementary file 5 — Additional file 5: Table S4. Univariable and multivariable analysis in different locations of gastric cancers. [file 12967_2019_1929_MOESM5_ESM.docx]

| **Table S4.Univariable and multivariable analysis in different locations of gastric cancers** | | | | | | | | |
| --- | --- | --- | --- | --- | --- | --- | --- | --- |
|  | **Univariable** | | | | **Multivariable** | | | |
|  |  |  |  | |  |  |  | |
|  | p-value | HR | 95%CI | | p-value | HR | 95%CI | |
| U |  |  |  | |  |  |  | |
| Age | 0.737 | 1.004 | 0.982 | 1.026 |  |  |  |  |
| Gender | 0.549 | 0.866 | 0.54 | 1.387 |  |  |  |  |
| Pathological classification | 0.162 | 1.175 | 0.937 | 1.474 |  |  |  |  |
| T stage |  |  |  |  |  |  |  |  |
| 1 | 0.018 |  |  |  |  |  |  |  |
| 2 | 0.925 | 0 | 0 | 1.58E+68 |  |  |  |  |
| 3 | 0.943 | 7.365 | 0 | 5.71E+24 |  |  |  |  |
| 4 | 0.93 | 11.952 | 0 | 9.20E+24 |  |  |  |  |
| N stage | 0.063 | 1.65 | 0.972 | 2.801 |  |  |  |  |
| M stage | ＜0.001 | 3.361 | 1.916 | 5.898 | 0.003 | 2.449 | 1.347 | 4.453 |
| pTNM |  |  |  |  |  |  |  |  |
| 1 | ＜0.001 |  |  |  |  |  |  |  |
| 2 | 0.134 | 4.862 | 0.616 | 38.401 |  |  |  |  |
| 3 | 0.017 | 11.228 | 1.545 | 81.584 |  |  |  |  |
| 4 | 0.001 | 29.224 | 3.824 | 223.366 |  |  |  |  |
| FOXP3 High vs Low | 0.001 | 2.171 | 1.385 | 3.404 |  |  |  |  |
| CD163 High vs Low | 0.776 | 1.067 | 0.684 | 1.664 |  |  |  |  |
| PD-L1 Pos vs Neg | 0.039 | 0.573 | 0.338 | 0.973 |  |  |  |  |
| CD3 High vs Low | 0.776 | 1.067 | 0.684 | 1.664 |  |  |  |  |
| CD8 High vs Low | 1.549 | 1.145 | 0.735 | 1.786 |  |  |  |  |
| FOXP3^high^PD-L1^neg^ | ＜0.001 | 2.623 | 1.666 | 4.13 | 0.002 | 2.123 | 1.309 | 3.443 |
|  |  |  |  |  |  |  |  |  |
| M |  |  |  | |  |  |  | |
| Age | 0.107 | 1.018 | 0.996 | 1.041 |  |  |  |  |
| Gender | 0.972 | 0.991 | 0.608 | 1.615 |  |  |  |  |
| Pathological classification | 0.26 | 1.145 | 0.904 | 1.45 |  |  |  |  |
| T stage |  |  |  |  |  |  |  |  |
| 1 | 0.007 |  |  |  |  |  |  |  |
| 2 | 0.896 | 0 | 0 | 1.97E+49 |  |  |  |  |
| 3 | 0.958 | 2.999 | 0 | 1.23E+18 |  |  |  |  |
| 4 | 0.869 | 30.331 | 0 | 1.23E+19 |  |  |  |  |
| N stage | ＜0.001 | 6.192 | 3.18 | 12.059 | ＜0.001 | 5.614 | 2.864 | 11.006 |
| M stage | ＜0.001 | 5.522 | 3.192 | 9.553 | ＜0.001 | 3.986 | 2.297 | 6.918 |
| pTNM |  |  |  |  |  |  |  |  |
| 1 | 0 |  |  |  |  |  |  |  |
| 2 | 0.858 | 18165.84 | 0 |  |  |  |  |  |
| 3 | 0.832 | 118737.689 | 0 |  |  |  |  |  |
| 4 | 0.819 | 292105.058 | 0 |  |  |  |  |  |
| FOXP3 High vs Low | 0.288 | 1.276 | 0.814 |  |  |  |  |  |
| CD163 High vs Low | 0.12 | 1.442 | 0.909 |  |  |  |  |  |
| PD-L1 Pos vs Neg | 0.005 | 0.443 | 2.252 |  | 0.075 | 0.594 | 0.336 | 1.053 |
| CD3 High vs Low | 0.125 | 0.703 | 0.448 |  |  |  |  |  |
| CD8 High vs Low | 0.029 | 0.606 | 0.387 |  | 0.015 | 0.567 | 0.358 | 0.896 |
| L |  |  |  | |  |  |  | |
| Age | 0.009 | 1.024 | 1.006 | 1.042 | 0.121 | 1.016 | 0.996 | 1.037 |
| Gender | 0.224 | 1.254 | 0.871 | 1.805 |  |  |  |  |
| Pathological classification | 0.001 | 1.329 | 1.12 | 1.578 | 0.101 | 1.161 | 0.971 | 1.387 |
| T stage |  |  |  |  |  |  |  |  |
| 1 | ＜0.001 |  |  |  | 0.025 |  |  |  |
| 2 | ＜0.001 | 0.231 | 0.109 | 0.489 | 0.004 | 0.324 | 0.15 | 0.702 |
| 3 | 0.822 | 0.822 | 0.675 | 1.64 | 0.384 | 1.22 | 0.779 | 1.91 |
| 4 | 0.001 | 1.893 | 1.307 | 2.74E+00 | 0.009 | 1.712 | 1.146 | 2.558 |
| N stage | ＜0.001 | 2.944 | 1.992 | 4.352 | 0.003 | 1.863 | 1.232 | 2.818 |
| M stage | ＜0.001 | 4.33 | 2.874 | 6.522 | ＜0.001 | 3.096 | 1.954 | 4.906 |
| pTNM |  |  |  |  |  |  |  |  |
| 1 | ＜0.001 |  |  |  |  |  |  |  |
| 2 | 0.001 | 4.27 | 1.763 | 10.345 |  |  |  |  |
| 3 | ＜0.001 | 11.07 | 4.796 | 25.55 |  |  |  |  |
| 4 | ＜0.001 | 25.525 | 10.549 | 61.758 |  |  |  |  |
| FOXP3 High vs Low | 0.39 | 0.863 | 0.616 | 1.208 |  |  |  |  |
| CD163 High vs Low | 0.28 | 1.204 | 0.86 | 1.686 |  |  |  |  |
| PD-L1 Pos vs Neg | 0.003 | 0.55 | 0.37 | 0.819 | 0.522 | 0.869 | 0.566 | 1.334 |
| CD3 High vs Low | 0.054 | 0.717 | 0.511 | 1.005 |  |  |  |  |
| CD8 High vs Low | 0.034 | 0.692 | 0.492 | 0.973 | 0.042 | 0.692 | 0.485 | 0.987 |

U:Upper; M:middle; L:low
