# Supplementary material for: Regulatory T cells and M2 macrophages present diverse prognostic value in gastric cancer patients with different clinicopathologic characteristics and chemotherapy strategies
Source: J Transl Med. 2019 Jun 7;17:192. doi: 10.1186/s12967-019-1929-9 (PMC6554965; doi:10.1186/s12967-019-1929-9)
Supplement: Supplementary file 6 — Additional file 6: Table S5. Univariable and multivariable analysis in different pathological classifications of gastric cancers. [file 12967_2019_1929_MOESM6_ESM.docx]

| **Table S5.Univariable and multivariable analysis in different pathological classifications of gastric cancers** | | | | | | | | |
| --- | --- | --- | --- | --- | --- | --- | --- | --- |
|  | **Univariable** | | | | **Multivariable** | | | |
|  |  |  |  | |  |  |  | |
|  | p-value | HR | 95%CI | | p-value | HR | 95%CI | |
| Pap |  |  |  | |  |  |  | |
| Age | 0.890 | 0.989 | 0.843 | 1.159 |  |  |  |  |
| Gender | 0.994 | 0.992 | 0.110 | 8.906 |  |  |  |  |
| Location | 0.633 | 0.777 | 0.277 | 2.185 |  |  |  |  |
| T stage |  |  |  |  |  |  |  |  |
| 1 | 0.686 |  |  |  |  |  |  |  |
| 2 | 0.641 | 0.019 | 0.000 | 339112.228 |  |  |  |  |
| 3 | 0.360 | 0.013 | 0.000 | 136.072 |  |  |  |  |
| 4 | 0.510 | 0.019 | 0.000 | 2582.841 |  |  |  |  |
| N stage | 0.249 | 69.871 | 0.051 | 96074.095 |  |  |  |  |
| M stage | 0.176 | 3.481 | 0.571 | 21.209 |  |  |  |  |
| pTNM |  |  |  |  |  |  |  |  |
| 1 | 0.989 |  |  |  |  |  |  |  |
| 2 | 0.954 | 0.000 | 0.000 |  |  |  |  |  |
| 3 | 0.966 | 0.000 | 0.000 | 2.241E+248 |  |  |  |  |
| 4 | 0.734 | 0.731 | 0.121 | 4.438 |  |  |  |  |
| FOXP3 High vs Low | 0.392 | 2.557 | 0.291 | 21.976 |  |  |  |  |
| CD163 High vs Low | 0.157 | 3.683 | 0.605 | 22.426 |  |  |  |  |
| PD-L1 Pos vs Neg | 0.291 | 0.393 | 0.069 | 2.227 |  |  |  |  |
| CD3 High vs Low | 0.447 | 0.034 | 0.000 | 210.381 |  |  |  |  |
| CD8 High vs Low | 0.658 | 0.043 | 0.000 | 46702.820 |  |  |  |  |
|  |  |  |  |  |  |  |  |  |
| Tub |  |  |  |  |  |  |  | |
| Age | 0.026 | 1.029 | 1.003 | 1.056 | 0.064 | 1.028 | 0.998 | 1.058 |
| Gender | 0.465 | 0.826 | 0.494 | 1.380 |  |  |  |  |
| Location | 0.237 | 0.838 | 0.625 | 1.123 |  |  |  |  |
| T stage |  |  |  |  |  |  |  |  |
| 1 | ＜0.001 |  |  |  | 0.008 |  |  |  |
| 2 | ＜0.001 | 0.097 | 0.030 | 0.312 | 0.006 | 0.182 | 0.054 | 0.615 |
| 3 | ＜0.001 | 0.304 | 0.148 | 0.623 | 0.014 | 0.393 | 0.186 | 0.829 |
| 4 | 0.265 | 0.702 | 0.377 | 1.308 | 0.237 | 0.667 | 0.341 | 1.306 |
| N stage | ＜0.001 | 4.373 | 2.493 | 7.670 | ＜0.001 | 2.942 | 1.623 | 5.331 |
| M stage | ＜0.001 | 5.504 | 2.734 | 11.080 | 0.032 | 2.249 | 1.072 | 4.722 |
| pTNM |  |  |  |  |  |  |  |  |
| 1 | ＜0.001 |  |  |  |  |  |  |  |
| 2 | ＜0.001 | 0.033 | 0.011 | 0.100 |  |  |  |  |
| 3 | ＜0.001 | 0.115 | 0.049 | 0.273 |  |  |  |  |
| 4 | 0.018 | 0.425 | 0.209 | 0.864 |  |  |  |  |
| FOXP3 High vs Low | 0.743 | 1.082 | 0.675 | 1.735 |  |  |  |  |
| CD163 High vs Low | 0.040 | 1.647 | 1.022 | 2.653 | 0.026 | 1.765 | 1.071 | 2.908 |
| PD-L1 Pos vs Neg | 0.002 | 0.462 | 0.281 | 0.759 |  |  |  |  |
| CD3 High vs Low | 0.627 | 1.124 | 0.701 | 1.804 |  |  |  |  |
| CD8 High vs Low | 0.197 | 0.729 | 0.450 | 1.179 |  |  |  |  |
| FOXP3^high^PD-L1^neg^ | 0.014 | 1.868 | 1.137 | 3.070 | 0.227 | 1.387 | 0.815 | 2.316 |

Pap:papillary adenocarcinoma; Tub:tubular adenocarcinoma

| **Univariable and multivariable analysis in different pathological classifications of gastric cancers** | | | | | | | | |
| --- | --- | --- | --- | --- | --- | --- | --- | --- |
|  | **Univariable** | | | | **Multivariable** | | | |
|  |  |  |  | |  |  |  | |
|  | p-value | HR | 95%CI | | p-value | HR | 95%CI | |
| Por |  |  |  | |  |  |  | |
| Age | 0.035 | 1.106 | 1.001 | 1.03 | 1.003 |  |  |  |
| Gender | 0.704 | 0.945 | 0.509 | 1.755 |  |  |  |  |
| Location | 0.167 | 0.874 | 0.721 | 1.058 |  |  |  |  |
| T stage |  |  |  |  |  |  |  |  |
| 1 | ＜0.001 |  |  |  | 0.01 |  |  |  |
| 2 | 0.012 | 0.151 | 0.034 | 0.664 | 0.023 | 0.178 | 0.04 | 0.787 |
| 3 | 0.983 | 0.993 | 0.502 | 1.962 | 0.86 | 1.063 | 0.537 | 2.105 |
| 4 | 0.008 | 2.149 | 1.221 | 3.781 | 0.004 | 2.308 | 1.297 | 4.108 |
| N stage | ＜0.001 | 2.209 | 1.544 | 3.161 | 0.009 | 1.633 | 1.129 | 2.362 |
| M stage | ＜0.001 | 4.291 | 3.025 | 6.088 | ＜0.001 | 3.149 | 1.979 | 5.012 |
| pTNM |  |  |  |  |  |  |  |  |
| 1 | ＜0.001 |  |  |  |  |  |  |  |
| 2 | 0.02 | 5.539 | 1.306 | 23.497 |  |  |  |  |
| 3 | ＜0.001 | 18.18 | 4.479 | 73.789 |  |  |  |  |
| 4 | ＜0.001 | 48.544 | 11.709 | 201.263 |  |  |  |  |
| FOXP3 High vs Low | 0.018 | 1.442 | 1.065 | 1.951 | 0.949 | 0.988 | 0.691 | 1.414 |
| CD163 High vs Low | 0.015 | 1.462 | 1.075 | 1.989 | 0.277 | 1.215 | 0.855 | 1.724 |
| PD-L1 Pos vs Neg | 0.088 | 0.713 | 0.483 | 1.052 |  |  |  |  |
| CD3 High vs Low | 0.002 | 0.617 | 0.458 | 0.832 | 0.246 | 0.814 | 0.574 | 1.153 |
| CD8 High vs Low | 0.01 | 0.677 | 0.502 | 0.913 | 0.499 | 0.883 | 0.617 | 1.266 |
|  |  |  |  |  |  |  |  |  |
| Sig+Muc |  |  |  |  |  |  |  | |
| Age | 0.048 | 1.031 | 1 | 1.062 | 0.973 | 1.001 | 0.968 | 1.035 |
| Gender | 0.857 | 0.945 | 0.509 | 1.755 |  |  |  |  |
| Location | 0.383 | 1.216 | 0.784 | 1.885 |  |  |  |  |
| T stage |  |  |  |  |  |  |  |  |
| 1 | 0.405 |  |  |  |  |  |  |  |
| 2 | 0.917 | ＜0.001 | 0 | 4.99E+63 |  |  |  |  |
| 3 | 0.942 | 6.878 | 0 | 1.86E+23 |  |  |  |  |
| 4 | 0.905 | 23.444 | 0 | 6.29E+23 |  |  |  |  |
| N stage | 0.001 | 4.565 | 1.922 | 10.843 | 0.001 | 4.713 | 1.937 | 11.467 |
| M stage | 0.021 | 2.625 | 1.16 | 5.939 | 0.002 | 3.675 | 1.582 | 8.537 |
| pTNM |  |  |  |  |  |  |  |  |
| 1 | 0.05 |  |  |  |  |  |  |  |
| 2 | 0.912 | 45881.742 | 0 | 5.73E+87 |  |  |  |  |
| 3 | 0.905 | 121031.685 | 0 | 1.51E+88 |  |  |  |  |
| 4 | 0.901 | 194820.975 | 0 | 2.43E+88 |  |  |  |  |
| FOXP3 High vs Low | 0.042 | 0.501 | 0.257 | 0.975 |  |  |  |  |
| CD163 High vs Low | 0.015 | 0.386 | 0.179 | 0.834 |  |  |  |  |
| PD-L1 Pos vs Neg | 0.112 | 0.536 | 0.248 | 1.157 |  |  |  |  |
| CD3 High vs Low | 0.228 | 0.675 | 0.375 | 1.279 |  |  |  |  |
| CD8 High vs Low | 0.155 | 0.386 | 0.179 | 0.834 |  |  |  |  |
| FOXP3^high^CD163^high^ | 0.029 | 0.206 | 0.05 | 0.852 | 0.011 | 0.154 | 0.036 | 0.656 |

Por:poor differentiated adenocarcinoma;Sig:signet ring cell addenocaecinoma;Muc:mucinous adenocarcinoma
