# Supplementary material for: Regulatory T cells and M2 macrophages present diverse prognostic value in gastric cancer patients with different clinicopathologic characteristics and chemotherapy strategies
Source: J Transl Med. 2019 Jun 7;17:192. doi: 10.1186/s12967-019-1929-9 (PMC6554965; doi:10.1186/s12967-019-1929-9)
Supplement: Supplementary file 11 — Additional file 11: Table S6. Univariable and multivariable analysis in different chemotherapy strategies of II–III gastric cancer. [file 12967_2019_1929_MOESM11_ESM.docx]

| **Table S6.Univariable and multivariable analysis in different chemotherapy strategies of II-III gastric cancer** | | | | | | | | |
| --- | --- | --- | --- | --- | --- | --- | --- | --- |
|  | **Univariable** | | | | **Multivariable** | | | |
|  |  |  |  | |  |  |  | |
|  | p-value | HR | 95%CI | | p-value | HR | 95%CI | |
| a+b+c |  |  |  | |  |  |  | |
| Age | 0.248 | 1.019 | 0.987 | 1.053 |  |  |  |  |
| Gender | 0.822 | 1.068 | 0.603 | 1.892 |  |  |  |  |
| Location | 0.825 | 0.96 | 0.671 | 1.374 |  |  |  |  |
| Pathological classification | 0.749 | 0.955 | 0.719 | 1.268 |  |  |  |  |
| T stage |  |  |  |  |  |  |  |  |
| 1 | 0.977 |  |  |  |  |  |  |  |
| 2 | 0.924 | 0.001 | 0 | 2.63E+57 |  |  |  |  |
| 3 | 0.928 | 8.433 | 0 | 1.11E+21 |  |  |  |  |
| 4 | 0.924 | 9.508 | 0 | 1.24E+21 |  |  |  |  |
| N stage | 0.139 | 2.414 | 0.751 | 7.762 |  |  |  |  |
| FOXP3 High vs Low | 0.051 | 1.807 | 0.997 | 3.273 |  |  |  |  |
| CD163 High vs Low | 0.004 | 2.419 | 1.321 | 4.429 |  |  |  |  |
| PD-L1 Pos vs Neg | 0.329 | 0.629 | 0.247 | 1.597 |  |  |  |  |
| CD3 High vs Low | 0.841 | 0.941 | 0.547 | 1.785 |  |  |  |  |
| CD8 High vs Low | 0.968 | 0.988 | 0.547 | 1.785 |  |  |  |  |
| FOXP3^low^CD163^low^ | 0.001 | 0.319 | 0.161 | 0.634 | 0.001 | 0.319 | 0.161 | 0.634 |
|  |  |  |  |  |  |  |  |  |
| a+b |  |  |  |  |  |  |  | |
| Age | 0.3 | 1.012 | 0.989 | 1.035 |  |  |  |  |
| Gender | 0.324 | 1.263 | 0.794 | 2.007 |  |  |  |  |
| Location | 0.339 | 0.871 | 0.655 | 1.157 |  |  |  |  |
| Pathological classification | 0.153 | 1.179 | 0.941 | 1.478 |  |  |  |  |
| T stage |  |  |  |  |  |  |  |  |
| 1 | 0.733 |  |  |  |  |  |  |  |
| 2 | 0.878 | 0.962 | 0.584 | 1.583 |  |  |  |  |
| 3 | 0.685 | 0.918 | 0.608 | 1.386 |  |  |  |  |
| N stage | 0.001 | 2.532 | 1.469 | 4.364 | 0.002 | 2.384 | 1.379 | 4.121 |
| FOXP3 High vs Low | 0.006 | 0.502 | 0.308 | 0.818 | 0.022 | 0.563 | 0.344 | 0.92 |
| CD163 High vs Low | 0.714 | 0.918 | 0.582 | 1.448 |  |  |  |  |
| PD-L1 Pos vs Neg | 0.489 | 0.842 | 0.517 | 1.371 |  |  |  |  |
| CD3 High vs Low | 0.317 | 0.797 | 0.511 | 1.243 |  |  |  |  |
| CD8 High vs Low | 0.003 | 0.506 | 0.323 | 0.793 | 0.006 | 0.533 | 0.34 | 0.837 |

a+b+c: fluorouracil 、cisplatin and paclitaxel, a+b: fluorouracil and cisplatin
